# Microbial Diversity in Bulk and Rhizosphere Soil of *Ranunculus glacialis* Along a High-Alpine Altitudinal Gradient

**DOI:** 10.3389/fmicb.2019.01429

**Published:** 2019-07-09

**Authors:** Nadine Praeg, Harald Pauli, Paul Illmer

**Affiliations:** ^1^Department of Microbiology, Universität Innsbruck, Innsbruck, Austria; ^2^Department of Integrative Biology and Biodiversity Research, Institute for Interdisciplinary Mountain Research and University of Natural Resources and Life Sciences Vienna, Austrian Academy of Sciences, Vienna, Austria

**Keywords:** rhizobiome, altitudinal gradient, biodiversity, bacteria, archaea, fungi, climate change, soil metagenomics

## Abstract

Serving as “natural laboratories”, altitudinal gradients can be used to study changes in the distribution of microorganisms in response to changing environmental conditions that typically occur over short geographical distances. Besides, rhizosphere zones of plants are known to be hot-spots for microbial diversity and to contain different microbial communities when compared with surrounding bulk soil. To discriminate the effects of altitude and plants, we investigated the microbial communities in the rhizosphere of *Ranunculus glacialis* and bulk soil along a high-alpine altitudinal gradient (2,600–3,400 m a.s.l.). The research area of this study was Mount (Mt.) “Schrankogel” in the Central Alps of Tyrol (Austria). Our results point to significantly different microbial diversities and community compositions in the different altitudinal belts. In the case of prokaryotes, environmental parameters could explain 41% of the total variation of soil communities, with pH and temperature being the strongest influencing factors. Comparing the effects derived from fraction (bulk vs. rhizosphere soil) and environmental factors, the effects of the roots of *R. glacialis* accounted for about one third of the explained variation. Fungal communities on the other hand were nearly exclusively influenced by environmental parameters accounting for 37.4% of the total variation. Both, for altitudinal zones as well as for bulk and rhizosphere fractions a couple of very specific biomarker taxa could be identified. Generally, the patterns of abundance of several taxa did not follow a steady increased or decreased trend along the altitudinal gradient but in many cases a maximal or minimal occurrence was established at mid-altitudes (3,000–3,100 m). This mid-altitudinal zone is a transition zone (the so-called alpine-nival ecotone) between the (lower) alpine grassland/tundra zone and the (upper) sparsely vegetated nival zone and was shown to correspond with the summer snow line. Climate change and the associated increase in temperature will shift this transition zone and thus, might also shift the described microbial patterns and biomarkers.

## Introduction

Mountain habitats are characterized by altitudinal gradients and dramatic climatic changes over short geographic distances, making them to ideal natural laboratories (Körner, 2007). Elevation gradients can be useful when investigating the effects of temperature, precipitation, solar radiation, wind, atmospheric pressure, and snow cover on biota (Körner, 2007; Bryant et al., 2008; Yuan et al., 2014; Wang J. T. et al., 2015; Siles and Margesin, 2016; Donhauser and Frey, 2018). In Europe, the Alps are the highest and most extensive mountain range system, comprising 2% of the total European area. Strong natural gradients and large spatial and temporal heterogeneities characterize the European Alpine landscape (Fischer et al., 2008). As in lowland ecosystems, the main parameters driving soil development in alpine environments are climate, biological factors, parent material, topography, and time (Donhauser and Frey, 2018). Similar to arctic environments, soil development is restricted by harsh climatic conditions and temperature and moisture affect the weathering type and rate. Due to the unfavorable conditions, soils develop incompletely and erosion often interrupts soil-forming processes. Thus, the formation of defined soil horizons is sometimes limited in alpine mountain soils, especially at higher altitudes (Donhauser and Frey, 2018). Comparable to arctic regions, low temperatures, which can drop below zero degrees Celsius, and long periods of snow cover limit biological processes and thus microbial life in mountain soils. However, soil microorganisms contribute substantially to biogeochemical cycles, such as carbon, nitrogen, and the phosphorous cycle. In the context of global change, alpine ecosystems have attracted much attention, largely because of their contribution to terrestrial carbon storage (Yan et al., 2015; Liu et al., 2016; Zhang K. et al., 2016; Wu L. et al., 2017). Global warming has increased the global mean temperature by up to 0.7°C in the last 100 years (Jones and Moberg, 2003), while in the Alps warming of 1.5°C has occurred over the same period (Böhm et al., 2001). This above-average warming, coupled with a high sensitivity of Alpine ecosystems, make the Alpine region one of the most vulnerable regions in Europe.

Soil microorganisms in mountainous areas must cope with harsh, oligotrophic, and nutrient-limited conditions. Coarse-textured soils with shallow horizons and a low water retention capacity do not protect against environmental stressors (e.g., temperature and moisture fluctuations). Despite the harsh conditions in (high) alpine ecosystems, mountain soils are places of considerable microbial diversities distinctly shaped by the environmental factors (Ciccazzo et al., 2014; Rime et al., 2015). Contrarily to the latitudinal gradients investigated in arctic ecosystems, studies in the alpine and nival zones of mountain environments are conducted along altitudinal transects, thereby addressing the influence of altitude-dependent climatic and biotic variations on microbial species distribution and functional diversity (Bryant et al., 2008; Fierer et al., 2011; Wang et al., 2012). Recent soil microbial ecology studies in mountain ecosystems have shown that there is no general altitudinal pattern in microbial diversity. Altitudinal patterns of alpha-diversities have either decreased with elevation (Bryant et al., 2008), increased with elevation (Wang et al., 2011), showed a maximum diversity at mid-elevations (Singh et al., 2012b), or no elevational pattern (Fierer et al., 2011; Wang et al., 2012). Unfortunately, it is difficult to compare the findings of these investigations because they either involve bacterial, archaeal and/or fungal communities, functional or genomic gene abundances, or phylogenetic relationships. Nevertheless, most of the studies have reported that environmental conditions related to altitude strongly influence microbial community structures (Donhauser and Frey, 2018). The main environmental drivers of bacterial community composition are carbon and nitrogen content, pH, nutrient and moisture contents, temperature, precipitation, and vegetation (Shen et al., 2013, 2015; Yuan et al., 2014; Rui et al., 2015). The influencing parameters for archaea are further extended to ammonium, nitrate, and potassium concentrations in soils (Singh et al., 2012a; Wang J. T. et al., 2015). Regarding fungi, pH, temperature, soil nutrients, and plant diversity were all contributing to the community structure (Lazzaro et al., 2015; Liu et al., 2015; Siles and Margesin, 2016).

The influence of plants on microorganisms remains understudied. Vascular plant cover and thus the amount of plant litter input were shown to decline with increasing altitude (Hofmann et al., 2016a), and plant litter is known an especially sheltered and energized habitat for microorganisms (Thies and Grossman, 2006). Regarding the succession of microorganisms in alpine ecosystems, microbial community structures are stabilized by the establishment of plants and are further shaped by interactions with the rhizosphere of these plants (Donhauser and Frey, 2018). More than 100 years ago, the importance of the interactions between microbes in the rhizosphere and plants were already recognized by Hiltner (1904) who defined the soil fraction influenced by rhizodeposition of exudates, mucilages, and sloughed cells as the rhizospheric area (Hiltner, 1904). Rhizosphere zones of plants can be hot-spots for microbial growth, abundance, and diversity as they offer habitats with increased nutrient availability. Recent climate warming has led to shifts in the distribution of vascular plants in high mountain environments across Europe (Pauli et al., 2012; Lamprecht et al., 2018), including the decline of plants adapted to high elevation in this study region (Pauli et al., 2007). Given the importance of plant communities for shaping bacterial and fungal distributions, the expected shifts in alpine plant communities, richness, and densities due to climate change could have large effects on bacterial and fungal community composition. The investigated Mount (Mt.) Schrankogel (Eastern Central Alps, Austria) is a master site of the Global Observation Research Initiative in Alpine Environments (GLORIA) and was established in 1994. Initially, the master site was founded to regularly monitor changes in the vegetation (Gottfried et al., 1998, 2011; Pauli et al., 1999, 2007), but was recently extended to studies targeting animals and microorganisms. The latter studies investigated the abundance and activity of archaea (total and methanogens) and bacteria, with a focus on nutrient cycling along this altitudinal gradient (Hofmann et al., 2016a,b; Winkler et al., 2018). The investigation of fungi in general and the community composition of both fungi and prokaryotes have not yet been part of the investigation program.

In the present study, we attempt to determine (i) whether the diversity and community compositions of soil prokaryotes and fungi vary across the elevation gradient. Considering the impact of plants, we (ii) assess the effect of the rhizosphere on prokaryotic and fungal communities compared to the effect of environmental and site-specific parameters. Finally, we (iii) identify specific biomarkers for each elevation zone and for the fractions bulk and rhizosphere.

## Materials and Methods

### Site Description

The study site of the investigation is Mt. Schrankogel, Eastern Central Alps, Stubai Alps, Tyrol, Austria (11°05′58′′E, 47°02′41′′N). Mt. Schrankogel (3,497 m) is the second highest mountain in the Stubai Alps. The northern and eastern sides of this mountain are surrounded by receding glaciers and the respective forelands. The altitudinal gradient investigated is located at the south-west facing slope of the mountain and covers altitudes ranging from 2,600 to 3,400 m above sea level (a.s.l.) (Supplementary Figure S1). The slope of the sampling sites is relatively uniform and ranges between 20 and 30°. Mt. Schrankogel is mainly comprised of siliceous bedrock (Hammer, 1929) and typical soil types are leptosols and cambisols. Mt. Schrankogel is a master site of the GLORIA (Global Observation Research Initiative in Alpine Environments) project and permanently installed GeoPrecision Mlog5W loggers (GeoPrecision, Germany) and Tinytag loggers (Gemini Data Loggers, UK) are used to measure soil temperatures at depths of 10 cm below ground. Important soil characteristics and sample site information are summarized in Table 1.

**Table 1:** Site descriptions for each altitude and physical and chemical soil characteristics including annual mean temperature (Temp), pH, dry matter content (DM), total carbon (C), total nitrogen (N), C/N ratio, and dissolved organic carbon (DOC) (shown are means, *n* = 3).

**Altitude (m)**	**Coordinates**	**Temp (^**°**^C)**	**pH**	**DM (g g^**−1**^)**	**Total C (mg g^**−1**^)**	**Total N (mg g^**−1**^)**	**C/N**	**DOC (μg g^**−1**^ DM)**	**Habitat**
2,600	47.033077° N, 11.096847° E	6.0	4.51	0.80	5.96	0.55	10.9	784.0	Snowbed with raw soil, scree, and boulders
	47.033077° N, 11.096847° E		4.25	0.79	5.84	0.42	13.8	838.1	
	47.033077° N, 11.096847° E		4.24	0.71	5.65	0.51	11.2	1115.9	
2,800	47.036946° N, 11.096869° E	4.86	4.40	0.71	8.01	0.77	10.5	1423.9	Snowbed with raw soil, scree, and boulders, crevice between boulders
	47.03684° N, 11.09527° E		4.32	0.75	6.42	0.62	10.3	1395.6	
2,900	47.038066° N, 11.091488° E	1.38	4.68	0.88	8.62	0.82	10.6	1270.3	Raw soil with scree, boulders and solid rock
	47.038066° N, 11.091488° E		4.56	0.73	4.54	0.40	11.4	448.8	
	47.038066° N, 11.091488° E		4.51	0.91	4.10	0.47	8.8	371.2	
3,000	47.039302° N, 11.092439° E	−0.37	4.70	0.91	4.54	0.41	11.1	280.2	Raw soil with scree, boulders, and solid rock
	47.039302° N, 11.092439° E		4.59	0.93	4.70	0.49	9.6	475.1	
	47.039302° N, 11.092439° E		4.59	0.86	4.34	0.41	10.7	503.1	
3,100	47.040371° N, 11.093758° E	−0.85	5.16	0.88	4.50	0.44	10.1	214.5	Raw soil with scree in solid rock habitat
	47.040371° N, 11.093758° E		5.04	0.88	5.09	0.49	10.5	496.8	
	47.040371° N, 11.093758° E		4.72	0.86	7.05	0.65	10.8	261.7	
3,200	47.041466° N, 11.094589° E	−3.41	4.30	0.93	13.43	1.08	12.5	299.1	Raw soil with scree in solid rock habitat
	47.041466° N, 11.094589° E		4.30	0.96	7.15	0.71	10.1	206.2	
	47.041466° N, 11.094589° E		4.36	0.92	5.86	0.53	11.1	251.4	
3,300	47.042685° N, 11.095657° E	−6.44	4.31	0.91	12.37	0.86	14.3	307.6	Raw soil with scree in solid rock habitat
	47.042685° N, 11.095657° E		4.46	0.90	4.33	0.33	13.2	594.3	
	47.042685° N, 11.095657° E		4.51	0.92	4.56	0.29	15.7	433.3	
3,400	47.043235° N, 11.097789° E	−3.45	4.35	0.93	8.58	0.58	14.7	313.1	Raw soil with scree in solid rock habitat, crevice with bryophytes/lichens in solid rock habitat
	47.043235° N, 11.097789° E		4.20	0.93	15.57	1.04	14.9	451.5	
	47.043235° N, 11.097789° E		4.28	0.85	21.07	1.55	13.6	264.5	

### Botanical Investigations and Choice of *R. glacialis* as a Test Plant

Previous botanical investigations of this altitudinal range have been reported by Gottfried et al. (1998) and Pauli et al. (1999). Alpine grasslands are dominated by *Carex curvula* and *Oreochloa disticha* which characterizes the alpine zones (2,600–2,900 m a.s.l.) whereas the nival zone (3,200–3,400 m a.s.l.) is dominated by *Saxifraga bryoides, Poa laxa*, and *Androsace alpina* (Gottfried et al., 1998). The zone in-between (3,000–3,100 m a.s.l.) is the so-called “alpine-nival ecotone” where the closed alpine vegetation is increasingly replaced by an open (nival) vegetation (Gottfried et al., 2011). To investigate the rhizospheric community structure, we chose *Ranunculus glacialis* (*Ranunculaceae*) as a test plant because it shows no altitudinal dependencies, yet occurs ubiquitously along the gradient.

### Soil Sampling

Plant and soil samples were collected on 2 days in mid-August 2016 at eight sites along the south-western slope of Mount Schrankogel ranging from 2,600 to 3,400 m, including three replicate sites at each altitude for bulk and rhizosphere soils, respectively (Supplementary Figure S1). Samples were collected at 100 m intervals, excluding samples from 2,700 m, as no *R. glacialis* samples could be found there. At 2,800 m, only two replicate plants were collected, meaning a total of 23 plant samples were collected overall. Soil samples (n = 23) were collected adjacent to the plant, being careful that the soil was root-free. The soil and plant samples were cooled, immediately transported to the laboratory, and stored at −20°C for further analysis. Soil samples were sieved to <2 mm for physical and chemical analyses.

### Physical and Chemical Soil Properties

Dry mass was determined by drying 5 g of the soil samples at 105°C overnight. The total carbon and total nitrogen contents of the soils were measured on a CN analyzer (Truspec CHN Macro, Leco, MI, USA) using oven-dried soil. Dissolved organic carbon (DOC) was quantified on a TOC-L analyzer (Shimadzu Co., Japan). Soil pH was measured in a CaCl_2_ [0.01 M] solution at a soil:solution ratio of 1:2.5 at room temperature. All physical and chemical soil properties were carried out in triplicate. Important soil properties and details of the site characteristics are summarized in Table 1.

### DNA Extraction

For DNA extraction, after shaking the plants gently to remove any loosely attached soil, the roots of *R. glacialis* were washed in 40 ml of sterile distilled water in an ultrasonic bath for 20 min. Bulk soil (300 mg) sieved to 2 mm was placed into 40 ml of sterile distilled water and shaken for 20 min. in an ultrasonic bath. The recovered soil slurries were centrifuged at 13,000 × g for 30 min. Subsequently, 35 ml of the clear supernatant was removed, and the remaining 5 ml were centrifuged again at 13,000 × g for 30 min. The clear supernatant was removed again. Finally, 1 ml of the centrifuged slurry was used for DNA extraction. DNA was extracted using the NucleoSpin® Soil Extraction Kit (Macherey-Nagel, Düren, Germany) according to the manufacturer's protocol (50 μl elution volume). The quality and quantity of the DNA extractions were controlled via UV/VIS spectrophotometry with NanoDrop 2000c™ (Thermo Fisher Scientific, Germany) and QuantiFluor® dsDNA Dye (Promega, Mannheim, Germany).

### Amplicon Sequencing

Prokaryotic and fungal microorganisms were identified by amplicon sequencing of the 16S rRNA and ITS2 genes, respectively. PCR amplification followed a two-step protocol. To amplify the V4 region of prokaryotes and the ITS2 fragment of fungi, the primer pairs 515f-806r (Caporaso et al., 2011) and gITS7-ITS4 (White et al., 1990; Ihrmark et al., 2012) were used, respectively. Archaea were sequenced separately using the primer pair Arch349f-Arch806r (Takai and Horikoshi, 2000). Amplicons were sequenced on the Illumina MiSeq v2 platform with 250 bp paired-end reads following Nextera library creation (Microsynth, Balgach, Switzerland). Sequence data were processed and further analyzed using the mothur software pipeline v.1.39.0 (64 bit executable) according to Standard Operating Procedures for paired-end sequencing (Schloss et al., 2009).

### Sequence Processing

Raw sequencing reads were demultiplexed, and adaptor and quality trimmed. Sequences were initially denoised to remove sequences that contained sequencing errors. In the case of fungi, ITS2 regions were extracted using the ITSx software (Bengtsson-Palme et al., 2013). Sequences with ambiguous reads [>6 homopolymers and a length of <240 bp or >270 bp (Archaea, Bacteria) or <150 bp (Fungi)] were removed. Chimeras were removed using the VSEARCH tool (Rognes et al., 2016). Unique prokaryotic sequences were aligned against the SILVA rRNA gene database (release 128) (Quast et al., 2013) using the kmer searching method classified using the RDP trainset reference database (Wang et al., 2007). Fungal sequences were pairwise compared to the fungal UNITE ITS database (Kõljalg et al., 2013). Binning of sequences to operational taxonomic units (OTUs) was performed using the OptiClust algorithm recently introduced in mothur at 97% identity. Rare OTUs with <5 reads were removed. All sequence data obtained in this study have been deposited in the National Center for Biotechnology Information (NCBI) Sequence Read Archive (SRA) and are accessible through the SRA accession number SRP176420. All other data are available from the corresponding author on request.

### Sequence Data Analysis and Statistics

Diversity and richness indices for prokaryotes and fungi were calculated using mothur (version 1.39) (Schloss et al., 2009). The Shannon-Wiener index was calculated to estimate species diversity and Chao1 was used as a richness estimator. Two-way ANOVAs were used to analyze the effects of altitude and fraction (bulk and rhizosphere soil) and their interaction on microbial alpha diversity and richness. Pearson's correlation analyses and multiple regression analyses assessed the relationship between microbial alpha diversity and environmental factors. Values of *p* ≤ 0.05 were considered significant. For further data analyses and additionally to allow direct comparisons of reads, the final OTU table was normalized by randomly subsampling to the lowest sequence reads obtained in a sample (91,690 reads and 10,984 reads for prokaryotes and fungi, respectively). Subsampling was proven not to differ significantly from the original data matrix by Mantel test. Non-metric multidimensional scaling (NMDS) was performed in R 3.4.2 (R-Core Team) using the package vegan (Oksanen et al., 2017) based on Bray-Curtis dissimilarity. Permutational multivariate analysis of variance (PERMANOVA) was used to model the influence of altitude and fraction on the Bray-Curtis dissimilarity matrix by applying the Adonis function in vegan (permutations = 999) (Oksanen et al., 2017). Biomarker taxa and OTUs being significantly differently abundant between the soil sites were analyzed with the LEfSe command using mothur and the public server at *usegalaxy.org* (Afgan et al., 2016). OTUs with a linear discriminant analyses (LDA) log score >3.7 were considered for interpretation. Redundancy analyses (RDA) was used to evaluate the relationship between microbial community structure and environmental variables (999 permutations). For RDA, response variables were log-transformed and to select variables for the RDA model, we used variation inflation factors (VIFs) to examine whether the variance of the regression coefficients is inflated by the presence of correlations with other environmental variables. Variation-partitioning analyses were performed to determine the relative importance of the environmental variables and their contributions to the microbial community composition were identified by partial redundancy analysis and hellinger transformed species data. Furthermore, generalized linear models with long-link transformation were used to test the influence of elevation and environmental properties on selected species groups.

Statistical analyses were carried out using mothur (version 1.39), R (version 3.4.2), R-Core Team [packages vegan (Oksanen et al., 2017), phyloseq (McMurdie and Holmes, 2013)] and Canoco (version 5.0) (Braak and Smilauer, 2012). In addition to sequence data analyses, Statistica 12.0 (StatSoft®) was used to perform multiple regression models to relate diversity and richness indices to environmental drivers and to calculate correlations of relative microbial abundances to selected environmental factors. Figures were produced using R (version 3.4.2), R-Core Team, partially applying ggplot2 (Wickham, 2016) and Microsoft Excel®.

## Results

### Overall Prokaryotic and Fungal Community Composition

**Figure 1:**
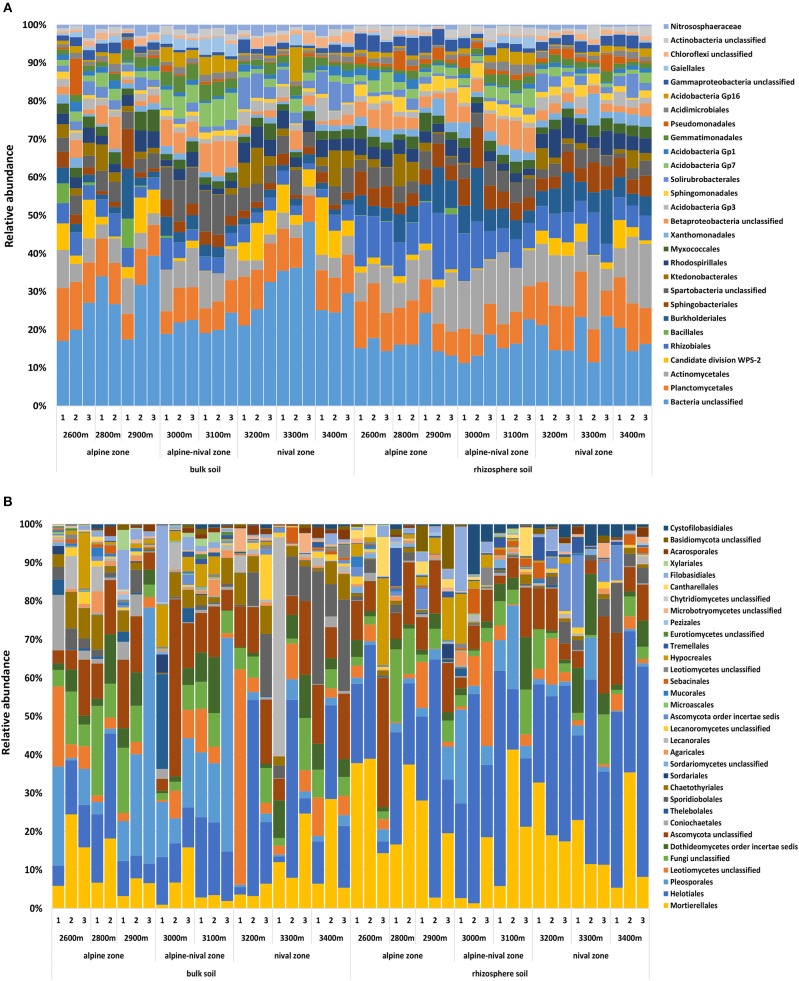
Community composition of prokaryotes **(A)** and fungi **(B)** on order level representing 90% of the most abundant prokaryotic and fungal orders in bulk soils and rhizosphere soils of *R. glacialis* along the altitudinal gradient (2,600–3,400 m, representing the alpine, alpine-nival, and nival zones). Numbers indicate the replicate sites at each altitude.

On average, Proteobacteria (21 and 34%), Actinobacteria (11 and 15%), Acidobacteria (11 and 9%), Planctomycetes (8% in bulk and rhizosphere), Verrucomicrobia (6% and 5%), Chloroflexi (4 and 3%), Candidate division WPS-2 (4 and 2%), and Bacteroidetes (3 and 6%) were the main representatives of the prokaryotic community in bulk and rhizosphere soils, respectively. The relative abundances of Acidobacteria, Actinobacteria, and Verrucomicrobia increased at mid-altitudes as did Gemmatimonadetes and Candidatus Saccharibacteria accounting for 2 and 0.8% relative abundance, respectively. In contrast, Chloroflexi, Proteobacteria and Candidate division WPS-2 decreased at mid-altitudes. No phylum increased or decreased linearly with increasing elevation and so the further information refers to order level as more information about the distribution of each group can be provided. Taxonomic information on phylum and class level for prokaryotes is given in Supplementary Figure S2. The taxonomic orders that together made up 90% of the total community within all bulk and rhizosphere soils are shown in Figure 1A. The most abundant orders were Planctomycetales (8.3%), Actinomycetales (7.4%), Rhizobiales (5.6%), Spartobacteria unclassified (4.1%), Burkholderiales (3.9%), Sphingobacteriales (3.3%), and Rhodospirillales (3.1%). Further orders included, among others, Acidobacteria Gp 1, 3, 7, and 16, Solirubrobacterales, Myxococcales, Ktedonobacterales, Gemmatimonadales, Xanothomonadales and Sphingomonadales. Archaea were represented by *Nitrososphaera* sp. only (probably *Nitrososphaera viennensis*) with a relative abundance of 1.3% and 0.5% in bulk and rhizosphere soils, respectively.

Ascomycota and Basidiomycota were the two most abundant phyla detected in fungal communities, accounting for 73% (63% in the rhizosphere) and 12% (13% in the rhizosphere), respectively. On average, the third most abundant phylum was Zygomycota, which represented 9% of the relative abundance in bulk soils and even 19% in the rhizosphere samples. Fungi that could not further be classified made up 6% and 5% in bulk and rhizosphere soils, respectively. At the phylum level, Ascomycota did not significantly increase or decrease with elevation while Basidiomycota significantly increased with increasing altitude and Zygomycetes decreased at mid-altitudes. The most abundant orders were Helotiales (Ascomycota) (21.7% on average), Mortierellales (Zygomycota) (13.7%), Pleosporales (Ascomycota) (8.0%), Dothideomycetes order incertae sedis (Ascomycota) (4.0%), Sporidiobolales (Basidiomycota) (3.6%), Hypocreales (Ascomycota) (2.4%), Chaetothyriales (Ascomycota) (2.2%), and Lecanorales (Ascomycota) (2.1%). Filobasidiales, Cystofilobasidiales, Pertusariales, Coniochaetales, Tremellales, Sebacinales, Cantharellales, Agaricales, Acarosporales, and Agyriales were among the orders representing <2% of the relative abundance. The orders that made up 90% of the total community within all bulk and rhizosphere soils are shown in Figure 1B. Taxonomic information on phylum and class level for fungi is given in Supplementary Figure S2.

### Community Patterns and Biomarkers of Prokaryotes and Fungi Along the Altitudinal Gradient

**Figure 2:**
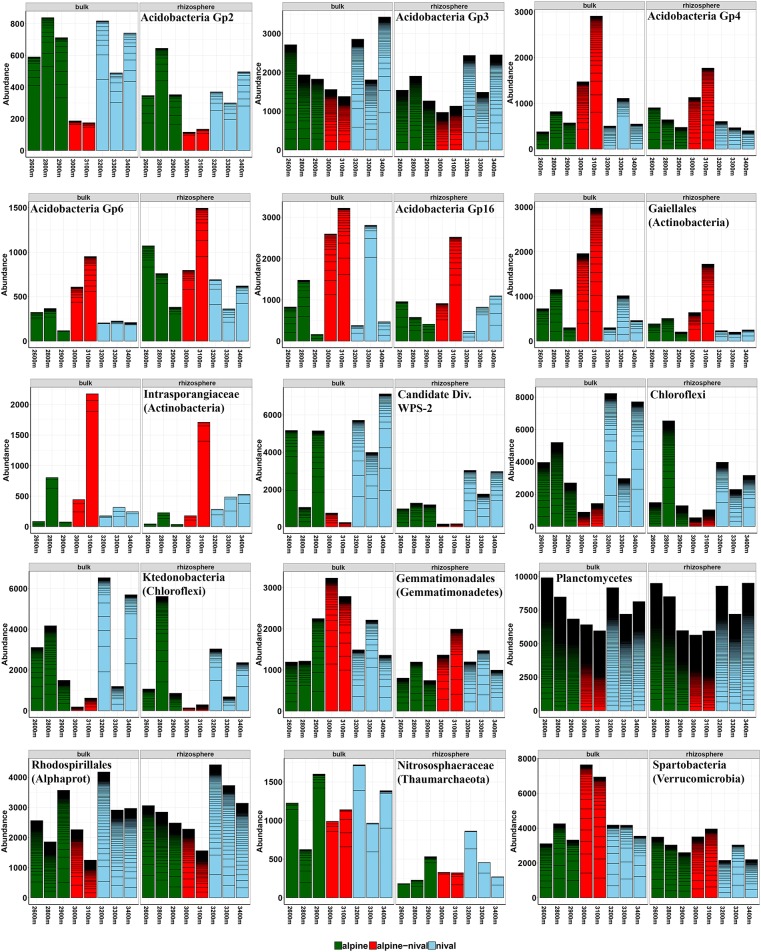
Selected abundance histograms of prokaryotic taxa that show significant changes according to the altitudinal gradient detected as biomarkers by LEfSe. Bars show absolute abundances (reads) of the selected taxa (*n* = 3). The abundances for OTUs within the selected taxa are stacked in order from greatest to least and are separated by thick black, stack lines. Elevations in the alpine zone (2,600–2,900 m) are indicated in green, alpine-nival zone (3,000–3,100 m) in red, and the nival zone (3,200–3,400 m) in blue. The left side of each single figure gives results derived from the bulk soils, the right side from the rhizosphere samples. The corresponding phylum, class or order of the selected taxon is given in parenthesis. Alphaprot: Alphaproteobacteria. Taxonomic groups are sorted alphabetically according to phylum.

**Figure 3:**
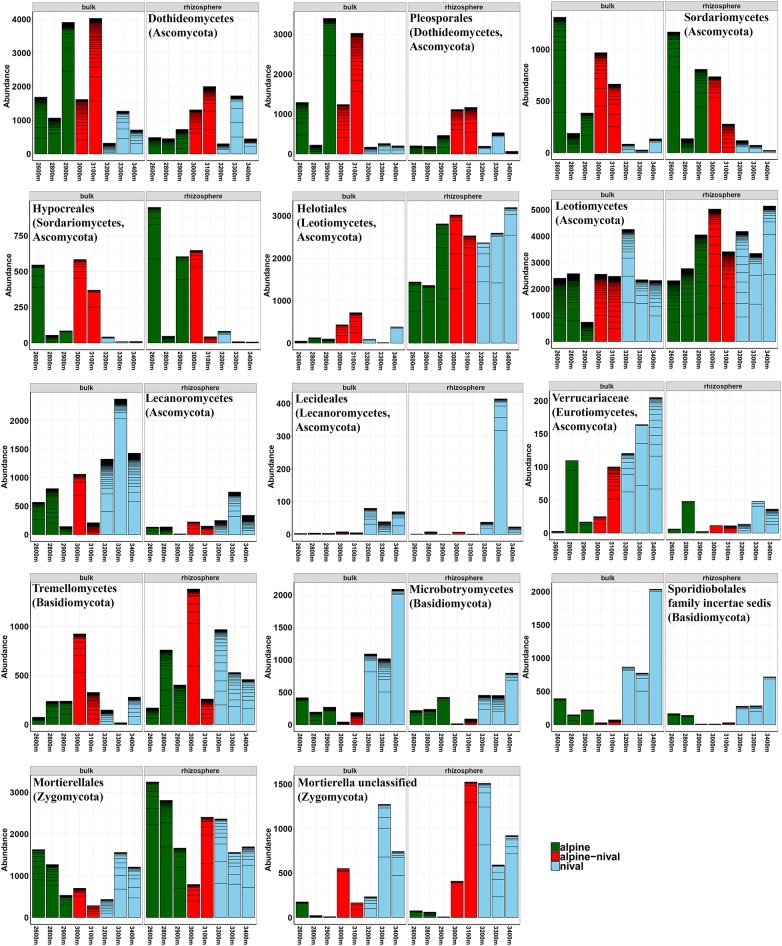
Selected abundance histograms of fungal taxa that show significant changes according to the altitudinal gradient detected as biomarkers by LEfSe. Bars show absolute abundances (reads) of the selected taxa (*n* = 3). The abundances for OTUs within the selected taxa are stacked in order from greatest to least and are separated by thick, black stack lines. Elevations in the alpine zone (2,600–2,900 m) are indicated in green, alpine-nival zone (3,000–3,100 m) in red, and the nival zone (3,200–3,400 m) in blue. The left side of each single figure gives results derived from the bulk soils, the right side from the rhizosphere samples. The corresponding phylum, class or order of the selected taxon is given in parenthesis. Taxonomic groups are sorted alphabetically according to phylum and follow the appearance in the text.

The influence of altitude was condensed to altitudinal zones that are well-described by environmental parameters and also community composition showed variations according to altitudinal zones. The grouping of altitudes according to ecological zones at Mt. Schrankogel has proven to be applicable for botanical (Gottfried et al., 1998) and microbiological investigations (Hofmann et al., 2016a) also facilitating interpretation. Thus, altitudes were categorized as alpine (2,600–2,900 m a.s.l.), alpine-nival (3,000–3,100 m a.s.l.), and nival (3,200–3,400 m a.s.l.) zones. The abundance profiles of prokaryotes along the elevation gradient showed four distinct patterns depending on the taxa observed. The abundances followed either a monotonically increase with altitude, a decrease with altitude, a hump-shaped decrease with a mid-altitude minimum, or a hump-shaped relationship with a mid-altitudinal peak, with the latter two being the most frequent patterns. Regardless of the fraction considered, the abundances of Acidobacteria Gp 4, 6, and 16 were highest at mid-altitudes (3,000 and 3,100 m) (Figure 2), partially leading to biomarkers for the alpine-nival zone. However, these increases in the acidobacterial groups at mid-altitude was accompanied by an increased abundance of members of Acidimicrobiales, Gaiellales, Intrasporangiaceae, Conexibacteraceae, and Cryptosporangiaceae (all Actinobacteria), Chitinophagaceae (Bacteroidetes), Gemmatimonadales, Hyphomicrobiaceae (Rhizobiales-Proteobacteria), and Spartobacteria (Verrucomicrobia) (selected taxa are shown in Figure 2). The increased abundances at mid-altitude were compensated by a significant decrease of Candidate division WPS-2 bacteria, the phylum Chloroflexi being mainly represented by Ktedonobacteria, and Planctomycetes (Figure 3). The order Rhodospirillales (Alphaproteobacteria) with Acetobacteraceae as the most abundant representatives showed a reduced occurrence at mid-altitude as well.

Altitude significantly influenced the total fungal community composition, as well as the bulk and rhizosphere fractions. In most cases, the pattern of taxa along the altitudinal gradient did not follow a linear (increased or decreased) scheme but varied highly between the sampled elevations. However, at the phylum level, Ascomycota were similarly abundant along the altitudinal gradient whereas Basidiomycota increased with increasing altitude (7 to 17% relative abundance in alpine and nival zones, respectively), especially in bulk soils. Some selected taxa are summarized in Figure 3. Dothideomycetes (Ascomycota) showed highest abundances at mid-altitudes. Likewise, the order Pleosporales (Dothideomycetes) increased at mid-altitude and showed a very reduced occurrence in the nival zone (3,200–3,400 m) (Figure 3). The Ascomycota order Sordariomycetes decreased steadily with increasing elevation (largely because of the order Hypocreales). Helotiales increased with altitude and was also highly significantly more abundant in the rhizosphere. A linear increase in abundance could be shown for Acarosporales (especially in the rhizosphere) along an increasing altitude. Lecideales and Pertusariales (both Lecanoromycetes-Ascomycota) were mostly limited to the nival zones. Similarly, in bulk soils, Verrucariaceae (Ascomycota) almost linearly increased with increasing altitude (Figure 3). The Basidiomycetes class Microbotryomycetes was highly abundant in the nival zone and moderately abundant in the alpine zone but not at mid-altitudes (2,900–3,100 m) (Figure 3). Zygomycota (mainly *Mortierella*) showed a lower abundance at mid-altitudes (2,900–3,100 m) (Figure 3) but several different OTUs occurred more or less exclusively at higher (3,300 and 3,400 m), mid (3,100–3,200 m), and lower (2,600–2,900 m) elevations, pointing to a very distinct adaptation of different *Mortierella* species to a specific altitude or altitudinal zone. Furthermore, several unclassified OTUs belong to *Mortierella* significantly increased with increasing elevation and had the highest relative abundances in the nival zones (Figure 3).

Microbial biomarker orders for each altitudinal zone were analyzed by LEfSe and are listed in Table 2. The most distinct biomarkers for the specific elevation range were Bacillales and Coniochaetales for alpine, Spartobacteria and Pleosporales for the alpine-nival, and Candidate division WPS-2 and Sporidiobolales for the nival zone. Obviously, very few microorganisms, both prokaryotes and fungi, are typical for the alpine zone. On the contrary, a couple of clades were shown to be typical for the alpine-nival zone and -as long as prokaryotes are concerned- also for the nival zone whereas only four orders of typical fungi could be identified for the nival zone. The relationship of the most abundant orders and biomarkers of prokaryotes and fungi with environmental parameters is summarized in Tables 3, 4. Biomarker orders for the altitudinal zones for the rhizosphere of *R. glacialis* are summarized in the Supplementary Table S1.

**Table 2:** Summary table showing prokaryotic and fungal biomarker species (at order level) in bulk soils for alpine, alpine-nival, and nival zones and for both fractions (bulk and rhizosphere) detected by LEfSe at LDA log score > 3.7.

	**PROKARYOTES**	**FUNGI**
**ALTITUDINAL ZONE**		
Alpine (2,600–2,900 m)	Bacillales	Coniochaetales
	Acidobacteria Gp2	
Alpine-nival (3,000–3,100 m)	Spartobacteria unclassified	Pleosporales
	Betaproteobacteria unclassified	Hypocreales
	Acidobacteria Gp7	Thelebolales
	Gemmatimonadales	Filobasidiales
	Acidobacteria Gp16	Xylariales
	Gaiellales	Capnodiales
	Desulfuromonadales	
	Acidobacteria Gp4	
Nival (3,200–3,400 m)	Bacteria unclassified	Sporidiobolales
	Candidate division WPS-2	Pertusariales
	Solirubrobacterales	Lecanorales
	Ktedonobacterales	Acasporales
	Acidobacteria Gp3	
	Chloroflexi unclassified	
	Nitrososphaeraceae	
	Ktedonobacteria unclassified	
**FRACTION**		
Bulk	Bacteria unclassified	Chaetothyriales
	Candidate division WPS-2	Lecanorales
	Spartobacteria unclassified	Coniochaetales
	Solirubrobacterales (Actinobacteria)	Agyriales
	Nitrososphaeraceae (Thaumarchaeota)	
Rhizosphere	Actinomycetales	Helotiales
	Rhizobiales	Mortierellales
	Burkholderiales	Cystofilobasidiales
	Sphingobacteriales	Tremellales
	Sphingomonadales	
	Xanthomonadales	

*Biomarkers are sorted by descending LDA score*.

**Table 3:** Generalized Linear Model showing the most abundant (>90% relative abundance) and relevant (biomarker) orders of prokaryotes and their dependencies on environmental parameters.

**Group**	**Temp**	**DM**	**pH**	**Ctot**	**Ntot**	**DOC**
Acidimicrobiales						
Acidobacteria Gp1	+^**^		–^*^			
Acidobacteria Gp2			–^*^			
Acidobacteria Gp3			–^**^			–^**^
Acidobacteria Gp4			+^***^			
Acidobacteria Gp6			+^***^			
Acidobacteria Gp7			+^**^			
Acidobacteria Gp16						
Actinobacteria unclassified			+^*^			
Actinomycetales	+^*^		+^**^			
Bacillales						+^*^
Bacteria unclassified	–^**^	–^*^	–^*^			
Betaproteobacteria unclassified			+^*^			
Burkholderiales		+^*^	+^*^			+^*^
Candidate division WPS-2			–^**^			
Candidatus Saccharibacteria	+^**^		+^*^			
Chloroflexi	–^*^		–^*^			
Desulfuromonadales			+^*^			
Flavobacteriales						
Gaiellales			+^**^			
Gammaproteobacteria unclassified			–^**^			
Gemmatimonadales			+^**^			
Ktedonobacterales			–^*^			
Myxococcales						–^*^
Nitrososphaeraceae			–^*^			–^*^
Planctomycetales			–^**^			
Pseudomonadales	+^*^			+^*^	–^*^	
Rhizobiales	+^**^					
Rhodospirillales			–^***^			–^*^
Solirubrobacterales			–^**^			
Spartobacteria			+^*^			
Sphingobacteriales		+^*^	+^*^			+^*^
Sphingomonadales			+^*^			
Xanthomonadales	+^***^	+^*^		+^**^	+^**^	

*Temp, annual mean temperature; DM, dry matter content; Ctot, total carbon; Ntot, total nitrogen; and DOC, dissolved organic carbon*.

*** *p < 0.001*,

** *p < 0.01*,

* *p < 0.05*;

+/– indicate positive (+) or negative (–) beta (b^*^) values.

**Table 4:** Generalized Linear Model showing the most abundant (>90% relative abundance) and relevant (biomarker) orders of fungi and their dependencies on environmental parameters.

**Group**	**Temp**	**DM**	**pH**	**Ctot**	**Ntot**	**DOC**
Acarosporales						
Agaricales						
Agaricomycetes order incertae sedis			–^*^	–^*^	+^*^	
Archaeosporales						+^*^
Ascomycota order incertae sedis						
Ascomycota unclassified						
Basidiobolales			–^*^	–^*^	+^*^	
Basidiomycota unclassified			+^*^			
Cantharellales						
Capnodiales						
Chaetothyriales						
Chytridiomycetes unclassified						
Coniochaetales						
Cystofilobasidiales						
Dothideomycetes order incertae sedis						
Eurotiales		+^*^				+^**^
Eurotiomycetes unclassified			+^*^			
Filobasidiales						
Fungi unclassified						
Helotiales						
Hypocreales						
Lecanorales						
Lecanoromycetes unclassified						
Leotiales			+^***^			
Leotiomycetes unclassified						
Microascales	+^**^		+^**^			
Microbotryomycetes unclassified						
Mortierellales				+^**^	–^***^	
Mucorales	–^*^					
Olpidiales			+^***^			
Pertusariales						
Pezizales						
Pleosporales						
Polyporales	–^*^					
Sebacinales						
Sordariales						
Sordariomycetes unclassified						
Sporidiobolales						
Thelebolales						
Tremellales			+^*^			
Xylariales			+^***^			

*Temp, annual mean temperature; DM, dry matter content; Ctot, total carbon; Ntot, total nitrogen; and DOC, dissolved organic carbon*.

*** *p < 0.001*,

** *p < 0.01*,

* *p < 0.05*;

+/– indicate positive (+) or negative (–) beta (b^*^) values.

### Community Patterns and Biomarkers for Prokaryotes and Fungi Influenced by the Rhizosphere

In general, a clear and significant influence of the rhizosphere of *R. glacialis* could be demonstrated, especially in connection with prokaryotes. On average, Proteobacteria and Actinobacteria increased from 21 to 34% and 11.4 to 14.5% relative abundance in the rhizosphere, respectively. In contrast, the phyla Acidobacteria and Candidate division WPS-2 decreased from 11.1 to 8.8% and 4.1 to 1.6% relative abundance in the rhizosphere compared with bulk soil, respectively. Verrucomicrobia and Chloroflexi decreased from 5.7 to 4.5% and 4 to 2.6% in the rhizosphere, respectively. Relative to the influence of environmental variables, the influence of fraction (bulk vs. rhizosphere) was of minor importance to the fungal community composition. Nevertheless, at the phylum level, the mean relative abundance of Ascomycota decreased from 72.6 to 63% relative abundance, while Zygomycota increased from 8.8 to 18.9% relative abundance in the rhizosphere. The large decrease of Ascomycota within the rhizosphere can be attributed to a significant decrease of Dothideomycetes, Eurotiomycetes, and Lecanoromycetes, but the class Leotiomycetes markedly increased.

The LEfSe-detected taxonomic biomarker orders (prokaryotes and fungi) in bulk and rhizosphere fractions are summarized in Table 2. Considering the fractions bulk and rhizosphere, a considerable number of orders differed highly significantly between the fractions (Table 2). The most significant biomarkers were Actinomycetales and Helotiales for the rhizosphere and Candidate division WPS-2 and Chaetothyriales for bulk soils. Biomarkers at OTU level for prokaryotes and fungi in bulk and rhizosphere soils are summarized in the Supplementary Tables S2, S3, respectively.

### Microbial (Beta-)Diversity of Bulk and Rhizosphere Soil Along the Altitudinal Gradient

**Figure 4:**
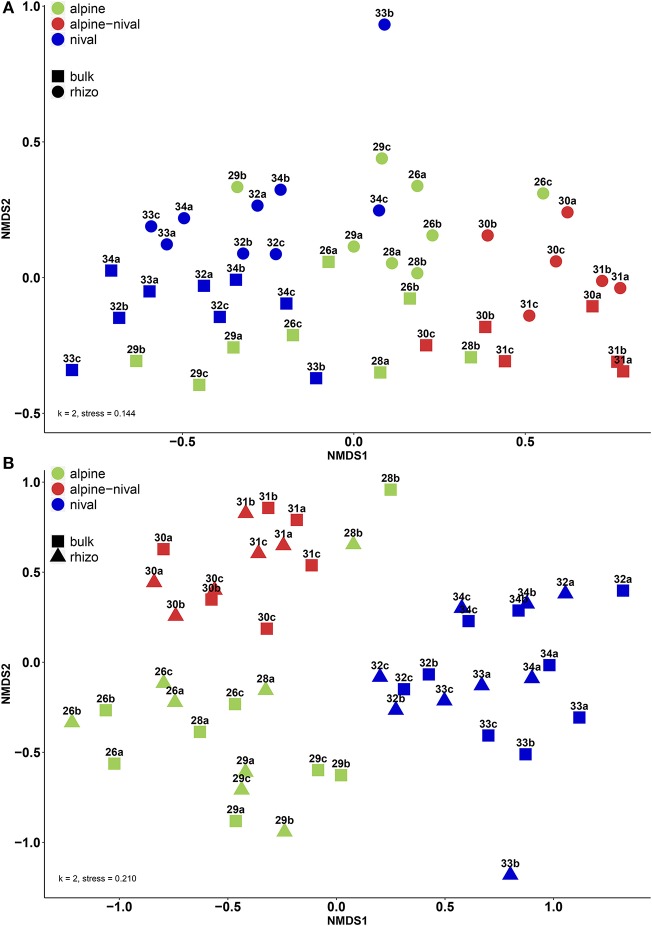
Non-metric multidimensional scaling (NMDS) plot displaying β-diversity by Bray-Curtis dissimilarities for **(A)** prokaryotic and **(B)** fungal communities. The samples are colored according to the altitudinal zones: green (alpine zone: 2,600–2,900 m), red (alpine-nival zone: 3,000–3,100 m), and blue (nival zone: 3,200–3,400 m). Numbers represent the respective altitudes, and letters indicate the replicates (e.g., 26a stands for the community at 2,600 m and replicate 1).

Figure 4 shows the β-diversity of prokaryotic (A) and fungal (B) communities using Bray-Curtis dissimilarities and non-metric multidimensional scaling (NMDS). For prokaryotes, the NMDS showed that altitude had a distinct influence on the community structure and that clustering according to the altitudinal zones could be achieved (Figure 4A). In contrast, the bulk and rhizosphere fractions did not lie very close together, neither among nor within the altitudes and within a specific altitude mainly differed with regard to NMDS2 (y-axis). Fungal communities led to an even more distinct clustering according to altitude. Contrarily to prokaryotes, bulk and rhizosphere soils clustered more closely within each altitude. The community structure of both, prokaryotes and fungi, were highly significantly influenced by altitude and fraction (Table 5). However, the *F*-value reflecting the influence of the fraction on fungal community is low, pointing to a minor importance of the fraction which supports the NMDS visualization. On the contrary, the *F*-value (as well as *R*^2^) reflecting the effect of the fraction on community structure of prokaryotes was very high proving the dominant overall effect of fraction for prokaryotes.

**Table 5:** Two-way permutational multivariate analysis of variance (PERMANOVA) (Adonis analysis) showing the effects of altitude and fraction (rhizosphere vs. bulk) and their interaction on microbial community structure.

**Factors**	***F***	***R*^**2**^**	***P***
**Prokaryotes**			
Altitude	3.649	0.069	0.002^**^
Fraction	6.634	0.125	0.001^***^
Altitude^*^Fraction	0.834	0.016	0.597
Residuals		0.791	
Total		1.000	
**Fungi**			
Altitude	4.077	0.083	0.001^***^
Fraction	2.137	0.044	0.001^***^
Altitude^*^Fraction	0.864	0.018	0.763
Residuals		0.856	
Total		1.000	

*F, F value by permutation; P, p-values based on 999 permutations*:

*** *p < 0.001*,

** *p < 0.01*.

**Figure 5:**
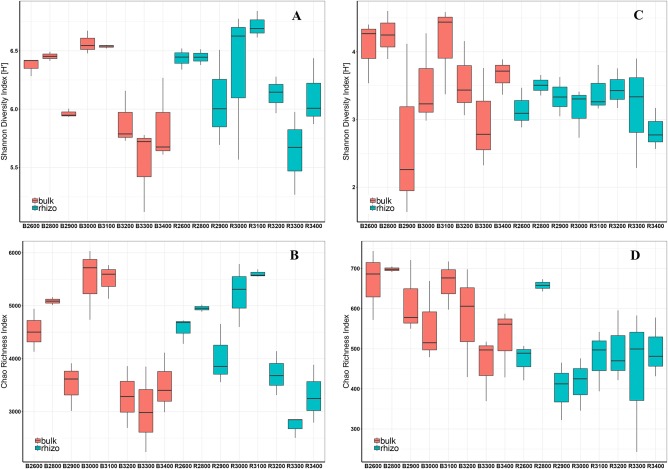
Shannon–Wiener Diversity Index (H') **(A)** and Chao Richness Index **(B)** of prokaryotic communities and Shannon-Wiener Diversity Index (H') **(C)** and Chao Richness Index **(D)** of fungal communities in all bulk (red) and rhizosphere (blue) soils along the altitudinal gradient. X-axis: B, bulk soils; R, rhizosphere soils. Numbers indicate the respective altitude (2,600–3,400 m) (e.g., B2600 = bulk soils at 2,600 m a.s.l.). Boxes represent 25–75% of values, black lines medians (*n* = 3), and whiskers 1.5 interquartile ranges.

Figure 5 shows the Shannon diversity index (Figure 5A) and Chao richness index (Figure 5B) for prokaryotes in bulk and rhizosphere soils along the altitudinal gradient. Altitude significantly influenced prokaryotic diversity. The Shannon diversity index for prokaryotes did not follow a monotonic in- or decrease along the elevation gradient but had a strong dependence on the respective elevation level. Irrespective of the very low diversity at 2,900 m, diversity reached its peak at an intermediate altitude of 3,000 and 3,100 m. At higher elevations, diversities decreased again and reached very low values at 3,300 and 3,400 m. Overall, microbial diversities did not differ significantly between bulk and rhizosphere soil, but rhizosphere soils showed higher diversities in the nival zones compared with the respective bulk soils (Figure 5A). Prokaryotic richness using the Chao index showed a comparable elevation-dependent pattern as the Shannon index and was significantly influenced by altitude and overall steadily decreased with increasing elevation (*r* = −0.513) (Figure 5B). Significant differences in species richness between bulk and rhizosphere soils were detected at 3,100 m only, and were comparable at the other altitudinal levels.

Shannon diversity index and Chao richness index for fungi in bulk and rhizosphere soils along the altitudinal gradient are shown in Figures 5C,D, respectively. Comparable to prokaryotic diversity, fungal diversity did not follow a monotonic decrease along the elevation gradient (Figure 5C). Neither altitude nor fraction (bulk and rhizosphere) significantly influenced fungal Shannon diversities. As with prokaryotes, the Shannon index of fungi was very low at an elevation of 2,900 m and reached higher levels at intermediate altitudes before it decreased again in the nival zone. Fungal species richness (Chao index) was significantly influenced by both, fraction and altitude. Overall, fungal species richness (Chao index) significantly decreased along an increased elevation. Considering the fraction differences, fungal species richness was significantly higher in bulk soils than rhizosphere soils (Figure 5D).

### The Effect of Environmental Variables on the Microbial Community Structure

**Figure 6:**
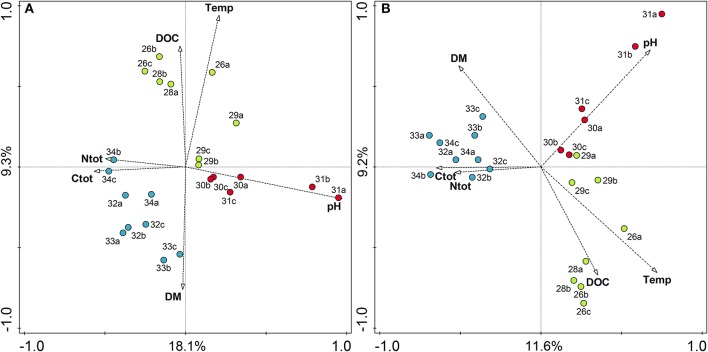
Redundancy analysis (RDA) biplot showing the relationship between environmental variables and prokaryotic **(A)** and fungal **(B)** communities along the altitudinal gradient color-coded by altitudinal zone. Elevations in the alpine zone (2,600–2,900 m) are indicated in green, alpine-nival zone (3,000–3,100 m) in red, and the nival zone (3,200–3,400 m) in blue. Percentage values on axes represent percentage variation of species-environmental relation explained by consecutive axes. Numbers represent the respective altitudes, and letters indicate the replicates (e.g., 26a stands for the community at 2,600 m and replicate 1). Temp, annual mean temperature; DM, dry matter content; Ctot, total carbon; Ntot, total nitrogen; and DOC, dissolved organic carbon.

RDA analysis with the prokaryotic OTU matrix of bulk soils and environmental variables (summarized in Table 1) showed that the soil parameters explained 41.4% of the total variation. Elevation was strongly correlated with temperature (*r* = −0.97, *p* < 0.001) and was excluded from the RDA analysis. By RDA analysis, we found that temperature and pH had the strongest effect on prokaryotic community composition. The biplot in Figure 6A shows sample sites as circles and environmental variables as arrows, demonstrating the important influence of pH and temperature and showing the clustering of the altitudinal zones according to species composition and influencing variables. The causal relationship of environmental parameters and groups were tested using generalized linear models (GLMs). Our analyses using GLMs with OTUs summarized on the order level revealed that especially pH, temperature, and DOC had significant effects on several orders (Table 3). For example, pH highly significantly affected Planctomycetales, Actinomycetales, Burkholderiales, Spartobacteria, Ktedonobacterales, Gemmatimonadales and a group of unclassified Gammaproteobacteria. Variation partitioning was performed to quantify the effects of fraction and environmental variables on community composition. In the case of prokaryotes, the effects of fraction and environmental variables were both significant. In bulk soils, environmental variables described 41.4% of the total variation in species data. Variation partitioning outlined environmental parameters and fraction (bulk vs. rhizosphere) to be responsible for 65.5% and 34.5% of the explained variation of the total species data, respectively.

Analogous RDA analysis of the fungal OTU matrix and environmental parameters showed that temperature and pH significantly influenced the fungal species composition, with an almost significant influence of total carbon content. RDA analysis showed that environmental parameters could explain 37.4% of the total variation. The biplot in Figure 6B again combines the information on species distribution and environmental variables (arrows). Variation partitioning for the total fungal OTU matrix with the factors fraction and environmental variables showed that the explained variation in the fungal community composition was almost completely described by environmental variables and only 2% of the variation was related to fraction. Thus, although the effect of the fraction on fungal community was found to be significant, the variation partitioning confirms the ordination in Figure 4, where the effect of altitude clearly exceeds the influence of the rhizosphere. Causal relationships between fungal species on order level and environmental parameters tested by GLMs are summarized in Table 4. Again, the influence of soil pH on the different groups, e.g., the orders Leotiales, Olpidiales, and Xylariales were highly significant. The influence of pH on the community is followed by the influence of the temperature, and then total organic carbon. For both, prokaryotes and fungi, RDA analyses showed that community composition in the alpine zone was influenced by higher temperature and higher dissolved organic carbon content, while the alpine-nival (mid-altitudinal) zone was influenced by (increased) pH, and the community composition in the nival zone was influenced by carbon and nitrogen content.

After summarizing the total community structure to common biodiversity indices, temperature and pH continued to be identified as significant determinants of prokaryotic diversity by our multiple regression models. Fungal diversity continued to be at least significantly influenced by temperature. The richness estimator Chao was significantly influenced by pH in the case of prokaryotes and temperature in the case of fungi.

## Discussion

In the present study, bacterial, archaeal, and fungal communities in bulk soil and the rhizobiome of *R. glacialis* were studied along a high-alpine altitudinal gradient (2,600–3,400 m a.s.l.) on Mount Schrankogel (Central Alps, Austria). One simple plant species (*R. glacialis*) was used for investigating the rhizobiome because it is one of the very few species growing above the entire altitudinal gradient and the use of only one species avoids species-specific effects. Studies on soil microorganisms in mountain ecosystems often lead to different results depending on the investigated groups, the investigated (sometimes too low) altitudes, climate zones, geologies, and vegetation. On the contrary, within our investigation a uniform geology, a uniform and consistent slope, and an elevation gradient lying entirely above the tree line characterize the present investigation site Mt. Schrankogel. The results of the present study show that prokaryotic and fungal communities are differently influenced by altitude, the rhizosphere, and environmental parameters like pH and are therefore partially discussed separately.

### Prokaryotic Community Patterns Influenced by Altitude

In the present investigation, the detected bacterial taxa included mainly Proteobacteria (primarily Alpha- and Betaproteobacteria), Actinobacteria, Planctomycetales, Acidobacteria, and Verrucomicrobia. Previous studies have reported these phyla as dominant in soils along elevation gradients in diverse mountainsides, including mountains on the Tibetan Plateau and Mt. Fuji in Japan (Singh et al., 2012b; Yuan et al., 2014; Wang J. T. et al., 2015; Yao et al., 2017). Verrucomicrobia and Acidobacteria are typically classified as oligotrophic microbes (Fierer et al., 2012; Cederlund et al., 2014) with the latter being associated with habitats with more recalcitrant carbon and low pH (Männistö et al., 2007; Lladó et al., 2017). In the present study, Acidobacteria showed distinct in- or decreases in the mid-altitudinal levels, depending on the specific group. Overall, Acidobacteria showed great pH sensitivity, but again depending on the specific group, the pH changes in the alpine-nival ecotone led to a significant in- or decrease of the respective acidobacterial group, finally leading to different Acidobacteria groups being biomarkers for the different altitudinal zones (Table 2). In other diverse studies, pH seems to especially influence the abundance, community, and diversity of Acidobacteria (Lauber et al., 2009; Chu et al., 2010), thus confirming our findings. Actinobacteria, the second most abundant phylum in our study, are generally widely distributed in various ecological environments and have been shown to survive under harsh conditions through the ability to form “mycelia” to reach water and nutrients and/or to produce spores to survive UV radiance or high salt concentrations (Dion, 2008; Rehákov et al., 2015). Furthermore, Actinobacteria play an essential role in the decomposition of soil recalcitrant matter and influence the weathering of mineral elements (Rehákov et al., 2015). The order Solirubrobacterales (Actinobacteria) could be established as a biomarker order for the nival zone, pointing to the importance of these organisms in the coldest and longest snow-covered areas of the mountain. Gaiellales (Actinobacteria), Gemmatimonadales, and Spartobacteria had increased relative abundances at mid-altitudes and were significantly influenced by pH as well, making all three taxonomic groups to biomarkers for the alpine-nival zone. Alpha- and Betaproteobacteria dominated the phylum Proteobacteria in bulk and rhizosphere soils along the gradient which goes in line with the findings of Yao et al. (2017). Alpha- and Betaproteobacteria are known to be associated with soils containing low C/N ratios (Yao et al., 2017) and Betaproteobacteria have additionally been shown to be increasing with increasing soil pH (Kim et al., 2014). This is consistent with our findings as Betaproteobacteria increased at mid-altitudes where soil pH was higher compared with the alpine and nival zones. The most dominant order within Betaproteobacteria at this elevation gradient was Burkholderiales which showed beside the dependency on pH a correlation with DOC content, in combination leading to the biomarker occurrence in the alpine-nival zone. Taxonomic groups that correlated with temperature were comparatively to pH less frequent but included, e.g., Chloroflexi which became a biomarker order for the nival zone as it increased with decreasing temperature. The decreasing DOC content along the gradient favored the occurrence of Acidobacteria Gp3 and Nitrososphaeraceae making these groups being typical for the nival zone as well.

The abundance of several other taxa did not show the typical hump-based peak at mid-altitude, but contrarily decreased or completely diminished in this zone. The Candidate division WPS-2 phylum could be identified as biomarker phylum for the nival zone. Its phylogenetic position remains unclear and ecological descriptions are rare but our results point to a strong (negative) correlation with pH. Planctomycetes made up > 8% of the relative abundance and showed great diversity, as indicated by the many black lines corresponding to the different OTUs in Figure 3. Most of the OTUs classified as Planctomycetes could not further be classified than at the phylum level. Given that currently only 26 planctomycetal genome sequences are known but on average and also in our study the phylum represents the fifth or fourth most abundant bacterial phylum in soils, the majority of sequenced Planctomycetes remains undescribed, and knowledge about planctomycetal physiology is rare (Ivanova et al., 2016; Wiegand et al., 2018). In our study, the abundance of Planctomycetes decreased at mid-altitude, and correlated with increasing soil pH (*r* = −0.72, *p* < 0.001). Generally, soil pH is a substantial soil characteristic that influences many biogeochemical processes affecting carbon, nitrogen, and phosphorous cycling (Rousk et al., 2010). pH influences soil microorganisms by modifying enzyme activity as well as controlling the availability of nutrients but also of toxic compounds like metals (Rousk et al., 2010; Ren et al., 2018). Thus, it cannot easily be predicted whether pH itself or pH-dependent soil properties are shaping microbial communities (Rousk et al., 2010).

Archaea were sequenced separately in addition to the use of general prokaryotic primers, but both sequencing results showed the single presence of *Nitrososophaera* species along the altitudinal gradient of Mt. Schrankogel. *Nitrososphaera* are capable of aerobic ammonium-oxidation and interestingly followed an increasing relative abundance pattern with increasing altitude and could be identified as a biomarker group for the nival zone. Relative abundance of Nitrososphaeraceae correlated with decreasing DOC content and increasing soil pH. Previous investigations have reported that *Nitrososphaera* could dominate the archaeal community in soils (Bates et al., 2010; Swanson and Sliwinski, 2013; Siles and Margesin, 2016) and were proven to be present in alpine and high-alpine environments as well (Zhang et al., 2009; Lamb et al., 2011; Siles and Margesin, 2016).

### Fungal Community Patterns Influenced by Altitude

Fungal community structure was mainly influenced by altitude, while fraction (bulk vs. rhizosphere) was of minor importance. Among the environmental variables measured, especially temperature and soil pH mostly affected the community structure of fungi. Albeit not significant the influence of total organic carbon (Ctot) was distinctly influencing the fungal community composition (*p* = 0.057) as well. Actually, fungal community composition often correlates with soil pH as shown also in previous investigations on mountainsides (Rousk et al., 2010; Zhang T. et al., 2016). Shen et al. (2014) and Coince et al. (2014) reported soil pH and temperature to be the most important predictor of fungal community structure in alpine soil in Northeast China, the French Alps, and Pyrenees (Coince et al., 2014; Shen et al., 2014), but, fungal community structure can be closely associated with soil nutrient status as well (Lauber et al., 2008) which confirms our findings. The fungal communities along the altitudinal gradient could be classified into three major groups according to the three main altitudinal zones, representing the alpine, the alpine-nival, and nival altitudinal belt. The only biomarker order for the alpine zone which is characterized by relatively high temperatures and concentrations of dissolved organic carbon was the order Coniochaetales, meaning that nearly no clade is specialized and adapted to this zone. The alpine-nival zone at mid-altitudes could be distinguished by the occurrence of five Ascomycetes orders while the nival zone harbored four biomarkers orders only. Correlations of fungal orders with environmental variables showed many dependencies on soil pH, temperature, and carbon content, while most of the biomarkers summarized at order level could not be correlated with soil properties. In general, fungal communities were dominated by Ascomycota, Basidiomycota, and Zygomycota. Biomarkers for the alpine-nival zone included Xylariales which correlated with the increasing pH at mid-altitudes. Overall especially the nival zone (altitudes above 3,100 m) contained an increased abundance of some taxa (e.g., Microbotryomycetes, Sporidiobolales, Lecideales) but lost groups like Sordariomycetes almost completely, explaining the lowest relative abundance of Ascomycota in the nival zone (still 67% of relative abundance). Besides, more rarely described psychrophilic species like *Pseudogymnoascus* sp. and the lichen-forming fungi *Bellemerea* sp. were present as well. *Pseudogymnoascus* sp. are cold-adapted Ascomycetes fungi, formerly found as predominant species in the glacier of the Qinghai-Tibet Plateau (Wang M. et al., 2015).

In bulk soils, Basidiomycota increased with increasing elevation from 7% relative abundance in the alpine, to 10% relative abundance in the alpine-nival, and 17% in the nival zone. The increase along the elevation is accompanied by decreasing DOC content, which is consistent with the findings of a previous study on Basidiomycota along an elevation gradient in China (Meng et al., 2013). Furthermore, the increase in Basidiomycota is also associated with the advent of yeasts associated with snow. Basidiomycetous yeasts are common psychrophiles, but because mycorrhiza are rare in alpine and arctic habitats (as there are no trees), only a few basidiocarp-forming fungi exist. Nevertheless, in our study three major Basidiomycetes orders were detected, including Sporidiobolales, Filobasidiales, and Cystofilobasidiales which mainly harbor typical psychrophilic and psychrotolerant Basidiomycetes (Frisvad, 2017). Typical cold-adapted fungi were *Rhodotorula* sp., a Microbotryomycetes yeast known to be psychrophilic and colonizing extreme (alpine) habitats (Margesin et al., 2007) and *Mrakia* sp., a Cystofilobasidiales yeast. *Mrakia* sp., and *Rhodotorula* sp. are both Basidiomycetes yeasts and members of the genera can secrete extracellular hydrolytic cold-active enzymes. Thus, the enabled mineralization of organic matter in cold environments makes cold-adapted yeasts a distinct contributor to biogeochemical cycling (Margesin et al., 2005; Thomas-Hall et al., 2010; Buzzini et al., 2012). Further typical psychrophilic representatives occurring on Mt. Schrankogel included *Cryptococcus* spp., thus especially the nival zone contained unique taxa making this zone particularly distinguishable from the lower zones. Depending on the observed fraction, Zygomycetes were the second (rhizosphere soils) or third (bulk soils) most abundant phylum. Many psychrophilic Zygomycetes have previously been found in cold ecosystems and different *Mortierella* species have been shown to be psychrophilic or psychrotolerant (Bergero et al., 1999; Robinson, 2001). In our study, we showed several different *Mortierella* spp. to be characteristic for the different alpine, alpine-nival, and nival zones along the south-western slope of Mt. Schrankogel, suggesting a high diversity of *Mortierella* and different preferred soil parameters.

### Effect of Altitude and Environmental Parameters on Microbial Community Structure and Diversity

In this study, prokaryotic diversity decreased with increasing altitude but with a peak at mid-altitudes (3,000–3,100 m) rather than in a linear pattern. Prokaryotic diversity was positively related to temperature and pH, leading to an increased diversity at highest pH values at mid-altitude and decreased diversity at lower pH values and lower temperatures toward the summit. These results confirm the findings of Siles and Margesin (2016), who associated higher diversities with increasing pH values when studying an elevation gradient in the Italian Alps. It is surprising that this also applies to our study, because the pH difference is only in the range of half a pH level. Studies dealing with microbial diversity and community patterns along elevation gradients have been performed in various mountainsides. Fierer et al. (2011) performed a comprehensive study in the eastern Andes and could not detect a significant trend for bacterial diversity along the studied elevation gradient (200–3,400 m) (Fierer et al., 2011). Bryant et al. (2008) studied the acidobacterial diversity and richness in the Rocky Mountains and showed that bacterial richness decreased monotonically from the lowest to highest elevations (2,460–3,380 m), and also correlated with temperature, pH, and total nitrogen. Soil pH is often reported as the key factor determining diversity patterns of microorganisms along elevation gradients (Singh et al., 2012b; Shen et al., 2013; Bartram et al., 2014; Zhang et al., 2015), and the results of our study point to the importance of soil pH. Moreover, elevation patterns were also suggested to be influenced by nutrient parameters. Lanzén et al. (2016) studied an altitudinal gradient in the Pyrenees and found an immense number of bacterial taxa correlating with the C/N ratio. Shen et al. (2015) showed linearly decreasing richness with increasing elevation and explained the pattern by decreasing dissolved organic carbon content (Shen et al., 2015). This also applies to our results comparing lowest and highest altitudes but again diversity and even richness peaked at mid-altitude. A possible explanation might be that the alpine-nival ecotone was shown to correspond with the summer snow line of the Eastern Alps (Gottfried et al., 2011) whereas the snowline is much higher in Tibetan or South-American mountains. Yao et al. (2017) showed that microbial community compositions studied along an elevation gradient at Changbai Mountain in Northeast China were shaped depending on vegetation and soil nutrient status leading to the use of oligotrophic-copiotrophic theory to explain the elevational distribution pattern of specific microbial taxa (Yao et al., 2017). Our study was performed at higher altitudes than that of Yao et al. (2017) including a gradient from 2,600 to 3,400 m and thus lying above the tree line. Depending on the microbial taxa considered, there are different dependencies with environmental factors, yet the pH value shows the strongest and most common influence (Tables 3, 4).

In our study, the alpha diversities of prokaryotes between the fractions (bulk and rhizobiome) did not differ significantly, but diversity in the rhizosphere increased at the highest altitudes (3,100–3,400 m). Interestingly, fungal diversity in the rhizosphere did not differ significantly according to altitude, but showed quite consistent and stabilized diversity along the entire gradient, whilst in contrast, fungal diversity in bulk soils increased or decreased at each altitude (Figure 5C). Thus, the rhizosphere of *R. glacialis* stabilized the fungal diversity and might have compensated for the spatial variability of soils and the effect of temperature. Regardless of the condensed diversity and richness indices, but analyzing total community structure, results showed that the most important soil features influencing microbial community composition and diversities were soil pH and temperature. These two key parameters are in return affecting many physiological and ecological parameters, and thereby influencing the growth and nutrient supply of microorganisms. Generally, microorganisms living in high-elevated ecosystems have the ability to survive and grow at low temperatures, and cope with restricted nutrient and water availability, as well as the viscosity of aqueous solutions and physical instability (D'Amico et al., 2006; Cary et al., 2010). Thus, only microbes with structural and functional adaptations can survive at these high altitudes. Changes in the lipid composition of the membrane, expression of cold-adapted enzymes, production of antifreeze proteins and cryoprotectants, and temperature-induced cold-shock and heat-shock responses help to overcome psychrophilic conditions (Margesin and Schinner, 1994; Lim et al., 2000; Feller and Gerday, 2003; Chintalapati et al., 2004; Gilbert et al., 2005; Buzzini et al., 2012). Besides temperature per se, water availability is another critical parameter for microbial activity. Liquid water is essential as a solvent for biochemical reactions and transport processes (Bakermans, 2008) and thus microbial activity in frozen systems is restricted to unfrozen amounts of water often containing a high amount of salts and exopolymeric substances (D'Amico et al., 2006). Therefore, prokaryotic and fungal growth in cold habitats requires the ability to grow at low water activity and xerophily might also be a prerequisite for psychrophily. However, despite these difficulties, mountainous environments are places of distinct prokaryotic and fungal communities. The investigated southwestern-slope of Mt. Schrankogel has previously been the focus of botanical investigations, leading to the description of three already mentioned ecological zones: alpine zone (2,600–2,900 m), alpine-nival zone (3,000–3,100 m), and the nival zone (3,200–3,500 m) (Pauli et al., 1999, 2007; Lamprecht et al., 2018). The elevation around 3,000 m a.s.l is considered as the alpine-nival ecotone (Gottfried et al., 1998) and to be one of the most distinct ecological transition zones in high mountain environments (Gottfried et al., 1998; Pauli et al., 1999, 2007), which was found to coincide with the elevation of the summer snow line at about 2,900 m (Gottfried et al., 2011). Besides the position of a permanent snowline and the turnover from alpine to nival plant species, soil pH values significantly increased from pH 4 to pH 5. Interestingly, this zone additionally reflects the point where the annual average temperature rises above the zero-degree Celsius line, and thus water can be present in its liquid form, leading to repeated freeze-thaw cycles and accelerated weathering. Therefore, microbial activities and nutrient cycling are stimulated, which was also confirmed by an increase in electrical conductivity and an increased amount of nutrient ions (e.g., ammonium, phosphorus) (data not shown).

### Microbial Communities Influenced by the Rhizosphere of *R. glacialis*

A key question of the study was to investigate if the rhizosphere soil community structure of *R. glacialis* differs from the community structure of the respective bulk soil and if the impact of plant is stronger than that of altitude and site-specific environmental parameters. In the Alps, at altitudes used for our investigation, the studied plant *R. glacialis* has to deal with conditions near the upper limits of higher plant life. Nevertheless, the rhizosphere led to the occurrences of especially prokaryotic but also fungal OTUs solely being abundant in the rhizobiome of *R. glacialis*. The rhizosphere is defined as the area directly influenced by root exudates. Root exudation depends on the plant's activity, and the released nutrients can act as stimulants and support nutrient sources for specific groups of microorganisms. Low temperatures are known to influence the activity of the plant roots, and thus might lead to reduced secretion of exudates and absorption of nutrients from soil. Overall, the community in the rhizobiome was comparable to that in bulk soils but significantly enriched with rhizosphere specific OTUs. Nevertheless, temperature and pH were again the best predictors of prokaryotic community composition in the rhizosphere along the altitudinal transect. Previous investigations dealing with the rhizosphere effect on microbial communities in harsh environments showed that plants alter the microbial community depending on the maturity status of the investigated plant (Tscherko et al., 2004; Edwards et al., 2006). On the contrary, Cui et al. (2019) investigated the rhizosphere soil microbial communities of *Abies fabri* along an altitudinal gradient below the timberline at the Tibetan Plateau and found that microbial communities varied significantly with altitude but not between the rhizosphere and bulk soil. Nevertheless, it was reported that despite the harsh environmental and physical conditions plants select specific communities (Ciccazzo et al., 2014). In our investigation, the influence of rhizosphere weakened the significant impact of altitude on prokaryotes, which was not the case for fungi. Considering prokaryotic communities, the rhizobiome of *R. glacialis* contained many biomarker taxa that were only abundant in the rhizosphere and several groups that were significantly more abundant in the rhizobiome compared with bulk soils. Thus, fraction could explain 34.5% of the explained variation in the community composition of prokaryotes. The rhizosphere of *R. glacialis* selected for the orders Actinomycetales, Rhizobiales, Burkholderiales, Sphingobacteriales, Sphingomonadales, and Xanthomonadales. Rhizobiales are well-known partners in plant-microbe interactions often providing beneficial nutrients, phytohormones, and precursors for essential plant metabolites (Delmotte et al., 2009) and many members are known to fix nitrogen (Garrity et al., 2015). King et al. (2012) reported a strong association of Burkholderiales with *Geum rossii* in high-alpine subnival zones in Colorado (King et al., 2012). The occurrences of the biomarker orders for the rhizosphere fraction of *R. glacialis* are also in line with the findings of Angel et al. (2016) who reported Rhizobiales, Shingobacteriales, and Sphingomonadales to be abundant in root-associated communities of various vascular plants in the Western Himalayas (Angel et al., 2016). Our reported increase in Proteobacteria in the rhizosphere fraction goes in line with another investigation of Fernández-Gómez et al. (2019) in the Andes who reported an increase of Proteobacteria in the rhizosphere of the studied *Poaceae* and *Caryophyllaceae* as a result of an increased abundance of the orders Rhizobiales, Sphingomonadales, and Burkholderiales. Sphingomonadales and Sphingobacteriales are known to metabolize diverse nutrients, including root exudates, and are frequently found on plant roots (Haichar et al., 2008). Furthermore, *Sphingomonas* harbor the nitrogenase gene, making this genus a potential root-associated diazotroph (Kämpfer et al., 1997). Besides, *Kaistia* sp. and *Bosea* sp. (both Alphaproteobacteria) were detected as biomarker OTUs for the rhizosphere of *R. glacialis* at Mt. Schrankogel (Supplementary Table S2) and were both found in the rhizosphere of the Andean plants as well (Fernández-Gómez et al., 2019). Interestingly, the genus *Kaistobacter* was identified as a plant-disease suppressor (Liu et al., 2016). Admittedly, Alphaproteobacteria contain many plant growth-promoting rhizobacteria (Pini et al., 2011). A previous investigation in the Sierra Nevada National Park (Spain) showed that the bulk and rhizosphere soil communities of a wild thyme species were structured according to the thermoclimatic zones along the altitudinal gradient studied (1,100–2,300 m) and changes differed whether communities were associated with the rhizosphere or studied in bulk soil (Pascual et al., 2018). Mainly Alpha- and Gamaproteobacteria had increased abundances in the rhizosphere compared with bulk soils (Pascual et al., 2018). In our study, the gradient was much higher (2,600–3,400 m) and we still found community differences not only due to altitude and altitude-dependent environmental parameters but according to the influence of the rhizosphere as well. This means that in the Himalayas as well as in the Alps all the way to the Andes plants build up similar rhizosphere communities, despite the difficult environmental conditions.

In the Alps, *R. glacialis* is known -like many Arctic plants- to be free of fungal colonization (Read and Haselwandter, 1981), which is likely a result of an asymmetric adaptation of mycorrhizal fungi to cold conditions as well as the inefficient nutrient acquisition (primarily N and P) of arbuscular mycorrhizal fungi at low temperatures (Kytöviita, 2005). Therefore, other parameters for the selection of fungi within the rhizosphere must be crucial. Mycorrhizal fungi along elevation gradients (ecto- and endomycorrhizal) have been comparatively well-studied, but free-living fungi are supposed to vary according to elevation and thus changing environmental variables as well. However, fungal communities within the rhizobiome of *R. glacialis* were characterized by an increased abundance of the class Leotiomycetes (largely as a result of the order Helotiales), Tremellales, Cystofilobasidiales, and Mortierellales. Interestingly, the order Helotiales was almost totally limited to the rhizobiome and almost no OTUs could be classified further, demonstrating much potential for the description of new species. Besides, various *Tetracladium* spp. (Supplementary Table S3) were typical for the rhizosphere of *R. glacialis* and has been shown to be associated with plants in previous investigations but to our knowledge not in cold and high-alpine soils (Xu et al., 2012; Turner et al., 2013). On the contrary, the abundance of Eurotiomycetes (mainly represented by Chaetothyriales and Verrucariales), Lecanoromycetes (mainly due to the order Acarosporales, Agyriales, and Lecanorales), Coniochaetales (Sordariomycetes) were largely or completely diminished in the rhizobiome which might be attributed to a repression of competitors. Nevertheless, and in contrast to prokaryotes, the community structure and diversity of fungi at least in bulk soil showed great deviations that made correlation and regression analyses with environmental parameters difficult. Once again this points to different and less predictable community changes of fungi compared with prokaryotes along with changing climate and soil properties. In contrast to lowland soils, mountain soils are characterized by a high local-scale heterogeneity that is caused by the complex topography associated with microclimatic regimes (Donhauser and Frey, 2018). One reason for the different responses of prokaryotic and fungal organisms might be the ability of fungi to form spores. These spores would be spread e.g., with wind and (rain) water more easily than vegetative cells.

In conclusion, altitude and thus temperature significantly shaped the prokaryotic and fungal community structure. The mid-altitudinal alpine-nival transition zone led to significantly increased or decreased abundances of several prokaryotic taxa, often following peak or trough response patterns at mid-altitudes, which could mostly be traced back to the pH difference in the alpine-nival zone compared to the lower alpine and higher nival zone where soil pH was lower. Fungal communities were also significantly affected by altitude, and altitude often led to the unique occurrence of fungi at the highest elevations in the nival zone. The microbial communities within the rhizosphere of *R. glacialis* were significantly different to that of bulk soils in the case of prokaryotes and the influence of the rhizosphere reduced the distinctness of altitude regarding the prokaryotic community composition. On the contrary, in the case of fungi the influence of the rhizosphere on community composition was not statistically significant. However, fungal diversity in the rhizosphere was highly stabilized compared with unstable fungal diversities in bulk soils. The soil itself influenced microbial communities predominantly, and the influence of *R. glacialis* was albeit significant less important compared with the influence of altitude-dependent soil characteristics. The discussed alpine-nival ecotone at mid-altitude of the investigated south-western slope of Mt. Schrankogel tends to follow the summer snow line. Thus, we predict that ongoing climate change will shift this ecotone upwards, which could have significant impact on microbial diversity and thus occurring taxa being specialized to this zone.

## Author Contributions

HP carried out the fieldwork. PI designed the study and helped with data interpretation and by contributing ideas. NP performed the sample processing, data analysis, data interpretation, and writing.

### Conflict of Interest Statement

The authors declare that the research was conducted in the absence of any commercial or financial relationships that could be construed as a potential conflict of interest.
