# Supplementary material for: Microbial Diversity in Bulk and Rhizosphere Soil of Ranunculus glacialis Along a High-Alpine Altitudinal Gradient
Source: Front Microbiol. 2019 Jul 9;10:1429. doi: 10.3389/fmicb.2019.01429 (PMC6629913; doi:10.3389/fmicb.2019.01429)
Supplement: Supplementary file 1 [file Table_1.docx]

**Supplementary Table S1:** Summary table showing prokaryotic and fungal biomarker species (at order level) in the rhizosphere of *R. glacialis* for the alpine, alpine-nival, and nival zone detected by LEfSe at LDA score > 3.7. Biomarkers are sorted by descending LDA score.

|  |  |  |
| --- | --- | --- |
| ALTITUDINAL ZONE | **PROKARYOTES** | **FUNGI** |
| alpine  (2,600–2,900 m) | Rhizobiales | Hypocreales |
|  | Gammaproteobacteria unclassified | Leotiomycetes order Incertae sedis |
|  | Pseudomonadales |  |
| alpine-nival  (3,000–3,100 m) | Actinomycetales | Pleosporales |
|  | Sphingobacteriales |  |
|  | Sphingomonadales |  |
|  | Flavobacteriales |  |
|  | Acidobacteria Gp6 |  |
|  | Actinobacteria unclassified |  |
|  | Candidatus Saccharibacteria unclassified |  |
|  | Methylophilales |  |
| nival  (3,200–3,400 m) | Burkholderiales | Sporidiobolales |
|  | Planctomycetales | Lecideales |
|  | Rhodospirillales |  |
|  | Xanthomonadales |  |
