# Supplementary material for: Microbial Diversity in Bulk and Rhizosphere Soil of Ranunculus glacialis Along a High-Alpine Altitudinal Gradient
Source: Front Microbiol. 2019 Jul 9;10:1429. doi: 10.3389/fmicb.2019.01429 (PMC6629913; doi:10.3389/fmicb.2019.01429)
Supplement: Supplementary file 2 [file Table_2.docx]

**Supplementary Table S2:** Summary table showing prokaryotic biomarker species in both fractions (bulk soil and rhizosphere soil of *R. glacialis*) detected by LEfSe at LDA score > 3.5. Taxonomic classification is given in parenthesis. Species are sorted by descending LDA score.

| FRACTION | Biomarker species |
| --- | --- |
| Bulk | *Nitrososphaera* spp. (Nitrososphaeraceae - Thaumarchaeota) |
|  | Bacteria unclassified (>5x) |
|  | Solirubrobacterales unclassified (Actinobacteria) |
| Rhizosphere | *Rhizobium* sp. (Rhizobiaceae - Alphaproteobacteria) |
|  | *Caulobacter* sp. (Caulobacteraceae - Alphaproteobacteria) |
|  | *Sphingomonas* sp. (Sphingomonadaceae - Alphaproteobacteria) |
|  | *Nakamurella* sp. (Nakamurellaceae - Actinobacteria) |
|  | *Rhizobacter* sp. (Pseudomonadaceae - Gammaproteobacteria) |
|  | Comamonadaceae unclassified (Comamonadaceae - Betaproteobacteria) |
|  | *Microbacterium* sp. (Microbacteriaceae - Actinobacteria) |
|  | *Kaistia* sp. (Rhizobiaceae - Alphaproteobacteria) |
|  | *Bosea* sp. (Rhizobiaceae - Alphaproteobacteria) |
|  | *Duganella* sp. (Oxalobacteraceae - Betaproteobacteria) |
|  | *Pedobacter* sp. (Sphingobacteriaceae - Bacteroidetes) |
|  | Methylophilales unclassified (Betaproteobacteria) |
|  | *Aminobacter* sp. (Phyllobacteriaceae - Alphaproteobacteria) |
|  | *Flavobacterium* sp. (Flavobacteriaceae - Bacteroidetes) |
|  | *Dokdonella* sp. (Rhodanobacteraceae - Xanthomonadales - Gammaproteobacteria) |
