# Supplementary material for: Microbial Diversity in Bulk and Rhizosphere Soil of Ranunculus glacialis Along a High-Alpine Altitudinal Gradient
Source: Front Microbiol. 2019 Jul 9;10:1429. doi: 10.3389/fmicb.2019.01429 (PMC6629913; doi:10.3389/fmicb.2019.01429)
Supplement: Supplementary file 3 [file Table_3.docx]

**Supplementary Table S3:** Summary table showing fungal biomarker species in both fractions (bulk soil and rhizosphere soil of *R. glacialis*) detected by LEfSe at LDA score > 3.5. Taxonomic classification is given in parenthesis. Species are sorted by descending LDA score.

| FRACTION | Biomarker species |
| --- | --- |
| Bulk | *Capronia* spp. (Herpotrichiellaceae - Ascomycota) |
|  | Leotiomycetes unclassified (Leotiomycetes - Ascomycota) |
|  | Leotiomycetes unclassified (Leotiomycetes - Ascomycota) |
|  | Leotiomycetes unclassified (Leotiomycetes - Ascomycota) |
|  | *Acarospora* spp. (Acarosporaceae - Ascomycota) |
|  | Helotiales unclassified (Ascomycota) |
|  | *Placynthiella* sp. (Lecanoromycetes - Ascomycota) |
| Rhizosphere | *Tetracladium* sp. (Helotiales family incertae sedis - Ascomycota) |
|  | *Tetracladium* sp. (Helotiales family incertae sedis - Ascomycota) |
|  | *Tetracladium* sp. (Helotiales family incertae sedis - Ascomycota) |
|  | *Tetracladium* sp. (Helotiales family incertae sedis - Ascomycota) |
|  | *Cryptococcus* sp. (Tremellales family incertae sedis - Basidiomycota) |
|  | *Dioszegia* spp. (Tremellales family incertae sedis - Basidiomycota) |
|  | *Haptocillium* spp. (Ophiocordycipitaceae - Ascomycota) |
|  | Ascomycota unclassified |
|  | Tremellomycetes unclassified |
