# Supplementary material for: Microbial Diversity in Bulk and Rhizosphere Soil of Ranunculus glacialis Along a High-Alpine Altitudinal Gradient
Source: Front Microbiol. 2019 Jul 9;10:1429. doi: 10.3389/fmicb.2019.01429 (PMC6629913; doi:10.3389/fmicb.2019.01429)
Supplement: Supplementary file 4 [file Image_1.pdf]

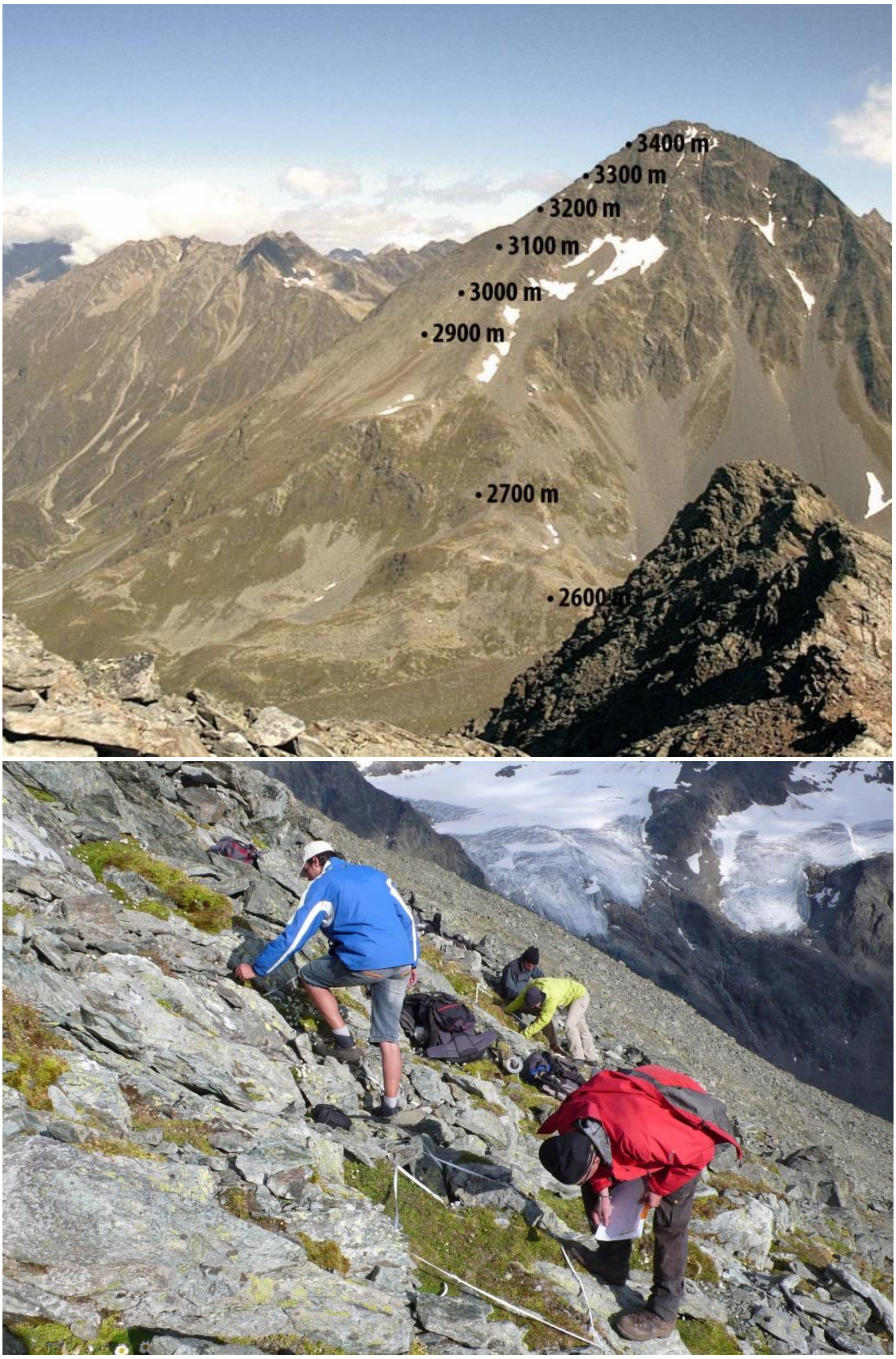

Supplementary Figure S1: Mt. Schrankogel (Eastern Central Alps, Austria, 3497 m a.s.l) and sampling altitudes on the south-western slope seen from the South (above) and impression of the sampling illustrating a quite uniform slope throughout the total altitudinal gradient (below).
