# Supplementary material for: Microbial Diversity in Bulk and Rhizosphere Soil of Ranunculus glacialis Along a High-Alpine Altitudinal Gradient
Source: Front Microbiol. 2019 Jul 9;10:1429. doi: 10.3389/fmicb.2019.01429 (PMC6629913; doi:10.3389/fmicb.2019.01429)
Supplement: Supplementary file 5 [file Image_2.pdf]

(A)

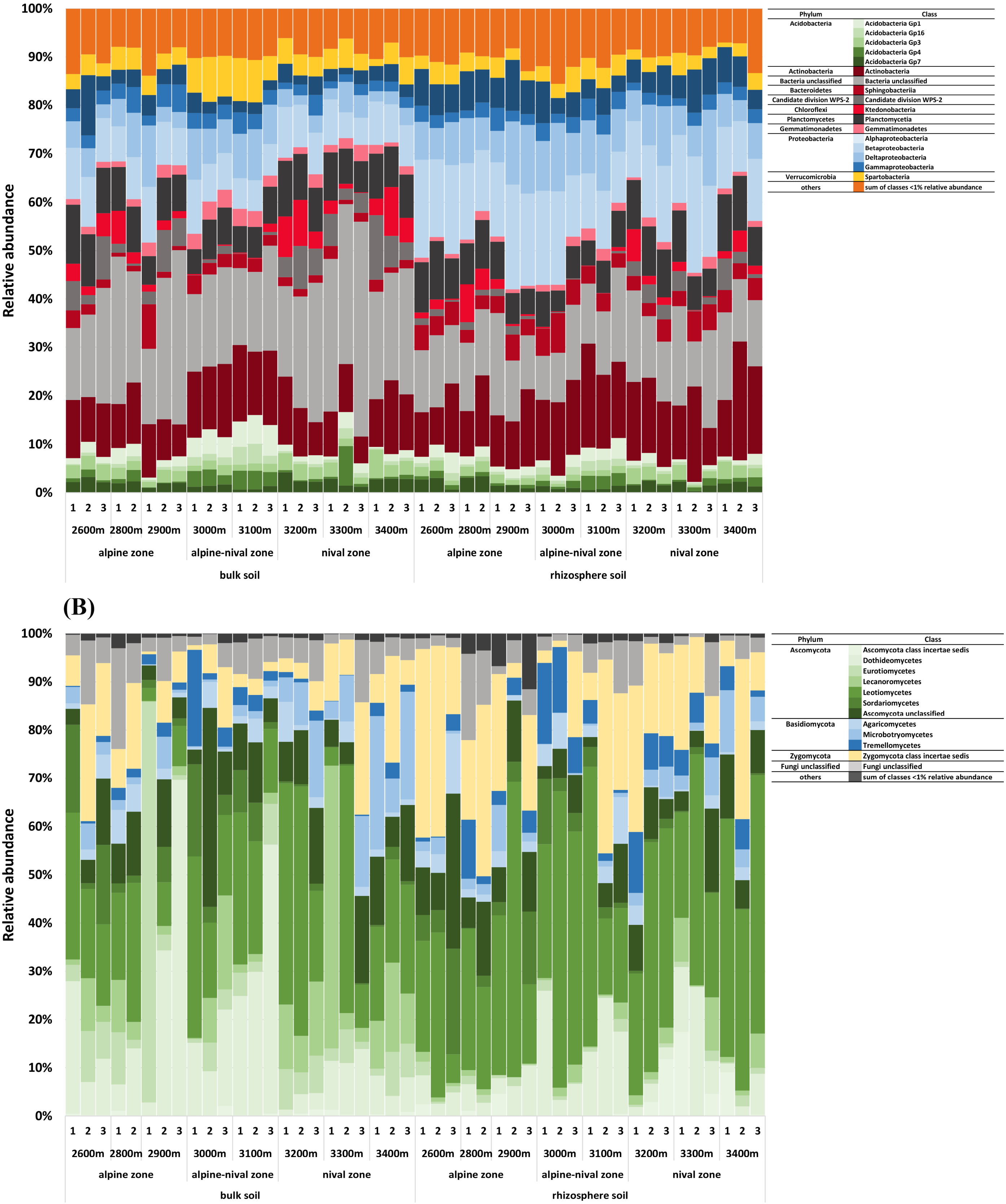

Supplementary Figure S2: Community composition of prokaryotes **(A)** and fungi **(B)** in bulk soils and rhizosphere soils of *R. glacialis* on class level along the altitudinal gradient (2,600–3,400 m a.s.l., representing the alpine, alpine-nival, and nival zones). Numbers indicate the replicate sites at each altitude.
